# Variability in drought gene expression datasets highlights the need for paired physiology and community standardization

**DOI:** 10.1093/plphys/kiaf653

**Published:** 2025-12-16

**Authors:** Robert VanBuren, Annie Nguyen, Rose A Marks, Catherine Mercado, Anna Pardo, Jeremy Pardo, Jenny Schuster, Brian St. Aubin, Mckena Lipham Wilson, Seung Y Rhee

**Affiliations:** Plant Resilience Institute, Michigan State University, East Lansing, MI 48824, United States; Department of Plant Biology, Michigan State University, East Lansing, MI 48824, United States; Department of Plant, Soil, and Microbiology, Michigan State University, East Lansing, MI 48824, United States; Plant Resilience Institute, Michigan State University, East Lansing, MI 48824, United States; Department of Horticulture, Michigan State University, East Lansing, MI 48824, United States; Plant Resilience Institute, Michigan State University, East Lansing, MI 48824, United States; Department of Plant Biology, University of Illinois, Urbana, IL 61801, United States; Plant Resilience Institute, Michigan State University, East Lansing, MI 48824, United States; Department of Plant Biology, Michigan State University, East Lansing, MI 48824, United States; Plant Resilience Institute, Michigan State University, East Lansing, MI 48824, United States; Department of Horticulture, Michigan State University, East Lansing, MI 48824, United States; Plant Resilience Institute, Michigan State University, East Lansing, MI 48824, United States; Department of Plant Biology, Michigan State University, East Lansing, MI 48824, United States; Plant Resilience Institute, Michigan State University, East Lansing, MI 48824, United States; Department of Plant Biology, Michigan State University, East Lansing, MI 48824, United States; Plant Resilience Institute, Michigan State University, East Lansing, MI 48824, United States; Department of Plant Biology, Michigan State University, East Lansing, MI 48824, United States; Plant Resilience Institute, Michigan State University, East Lansing, MI 48824, United States; Department of Plant Biology, Michigan State University, East Lansing, MI 48824, United States; Department of Horticulture, Michigan State University, East Lansing, MI 48824, United States; Plant Resilience Institute, Michigan State University, East Lansing, MI 48824, United States; Department of Plant Biology, Michigan State University, East Lansing, MI 48824, United States; Department of Plant, Soil, and Microbiology, Michigan State University, East Lansing, MI 48824, United States; Department of Biochemistry and Molecular Biology, Michigan State University, East Lansing, MI 48824, United States

## Abstract

Physiologically relevant drought stress is difficult to apply consistently, and the heterogeneity in experimental design, growth conditions, and sampling schemes makes it challenging to compare water deficit studies in plants. Here, we reanalyzed hundreds of drought gene expression experiments across diverse model and crop species and quantified the variability across studies. We found that drought studies are surprisingly incomparable, even when accounting for differences in genotype, environment, drought severity, and method of drying. Many studies, including most *Arabidopsis* (*Arabidopsis thaliana*) work, lack high-quality phenotypic and physiological datasets to accompany gene expression, making it challenging to assess the severity or consistency of water deficit stress events. To help address this, we developed supervised learning classifiers that can distinguish RNAseq samples that likely experienced drought stress. While not a substitute for direct measurements, these classifiers may aid in interpreting existing datasets and assessing drought severity in studies lacking physiological metadata. Together, our analyses highlight the importance of paired physiological data to quantify stress severity for reproducibility and future data analyses.

## Teaser

A comparative analysis of plant drought studies reveals widespread variability and highlights the need for paired physiological data to improve consistency and interpretability.

## Introduction

Drought, increasingly prevalent in both natural and agricultural landscapes, is escalating in frequency and severity due to the dynamic climate. This trend has spurred the development of an extensive and increasingly interdisciplinary research community focused on understanding plant adaptation to water-limited environments (Osmolovskaya et al. 2018; Ekundayo et al. 2022). Meteorologically, drought manifests as drier than normal conditions, but its physiological impact on plants varies based on the duration, severity, and timing of the stress events, alongside local soil and habitat conditions (Tardieu 2012; Gupta et al. 2020). Mild, infrequent drought events may result in only slight reductions in photosynthesis and growth, often without significant impacts on biomass or yield. In contrast, recurrent or severe episodes of drought may cause unrecoverable damage or even plant death (Farooq et al. 2009). Central to understanding and engineering drought resilience is the ability to apply consistent, physiologically relevant, and reproducible stress events across scales (Großkinsky et al. 2015). Such standardization is necessary to develop a community framework that allows for comparison and expansion of previous experiments.

Water deficit responses likely evolved during terrestrialization, and they have been continually refined, repurposed, and diversified to enable plants to colonize virtually every biome (Bowles et al. 2021). Resilience to drought is an emergent phenotype involving the synchronization of numerous physiological and genetic processes, and diverse lineages of plants have evolved numerous adaptations to avoid, escape, and tolerate water deficits (Turner 1986; Chaves and Oliveira 2004; Artur and Kajala 2021). Different plant lineages, populations, or even individual genotypes use combinations of these strategies to tolerate water limitations (Farooq et al. 2009; Verslues and Juenger 2011; Basu et al. 2016). The genetic mechanisms underlying responses or tolerance to drought stress are highly complex and involve the activation of hundreds to thousands of genes that collectively enable resilience to water deficit. Most drought-related pathways were initially discovered and characterized in the model plant *Arabidopsis*, but core regulatory, biochemical, and physiological responses are broadly conserved across green plants. Although tolerance varies significantly within and across species, a core transcriptional machinery underlying general drought responses has been observed, regardless of innate sensitivity (Chen et al. 2021; Pardo et al. 2023; Marks et al. 2024). Advancing our understanding of these mechanisms will require integrating transcriptomic datasets that span mild to severe drought stress across diverse genotypes, tissues, and environmental conditions and are coupled with informative and well-annotated physiological data on plant water status. The genetic basis of adaptations to water deficit is an active and exciting area of plant science research, and numerous important research gaps still remain (Eckardt et al. 2023; Verslues et al. 2023).

One promising approach to closing the knowledge gaps in understanding the genetic basis of drought adaptation is using large-scale omics technologies. Numerous large-scale datasets have been collected across diverse plant lineages to study the effects of drought stress. Some studies have measured physiological responses in naturally water-limited environments (Danilevskaya et al. 2019; Groen et al. 2022; Pardo et al. 2023), but most use simulated drought events under controlled or semicontrolled conditions to induce water deficit responses. Simulated drought studies range in scale and severity from large rainout shelters withholding water from thousands of plants in an ecological or agricultural setting to agar plates containing solutes to lower water potential. Each of these approaches has benefits and drawbacks related to cost, consistency, and accuracy of applying drought. Individual labs utilize radically different experimental approaches, growth conditions, and sampling schemes for drought assays, and these added variables mask emergent properties of an already complex phenotype. A major challenge for cross-species analysis is finding comparable biological datasets with similar design, implementation, and sampling.

Here, to evaluate the comparability and reusability of drought gene expression data, we compared public datasets across labs and experiments and searched for patterns that delineate drought and control conditions. We first focused on data from the model plant *Arabidopsis thaliana* and then expanded our analyses to include 5 additional model and crop species with the most published drought data. We found that drought gene expression data are more variable compared to heat stress, and many studies lack basic physiological data to assess the magnitude or even presence of water deficit stress. Our analyses highlight the importance of clearly measuring and reporting plant water status to improve interpretation and integration of drought transcriptomic data across studies.

## Results

### Variability of gene expression datasets on drought in *Arabidopsis*

Plant responses to drought are a well-studied topic. There are ∼34,000 articles in PubMed related to drought stress in plants, and this wealth of knowledge has uncovered numerous phenotypes, pathways, and genes underlying responses to water deficit (Gupta et al. 2020). Most of our understanding of drought responses at the molecular genetic level is based on work in *Arabidopsis*, including over 100 studies surveying genome-wide gene expression (RNAseq) changes under water deficit across different accessions, environmental conditions, and drought treatments (Supplementary Table S1). Collectively, these datasets have been incorporated into public gene expression atlases, coexpression networks, and other tools that are broadly used by the plant science community to understand which genes and pathways underlie drought responses (Lamesch et al. 2012; Klepikova et al. 2016). However, drought experiments vary wildly in the degree, severity, and implementation of water deficit. Many experiments are analyzed in isolation and arrive at independent conclusions. The choice of which drought experiments to reference for future studies can drastically alter hypothesis generation and inference of biological function. This raises a fundamental question, how comparable are drought studies across different experiments?

**Figure 1.**
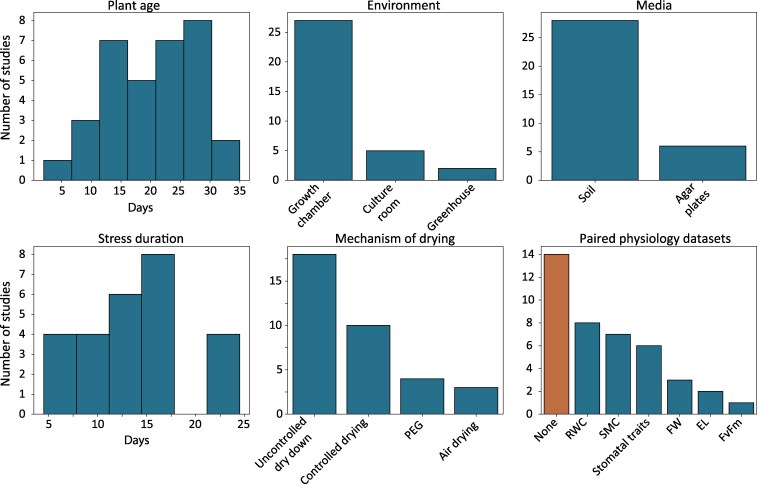
Summary of *Arabidopsis* drought gene expression metadata. Metadata was collected for the 36 BioProjects with associated publications. The top row shows a histogram of developmental stage or age of plants (in days) at sampling, the environment where studies were conducted, and the media plants were propagated in. The bottom row shows a histogram of the duration of water deficit stress, mechanism of drying, and paired physiology data. Studies using PEG or air drying are not plotted in the stress duration graph, as experiment times ranged from 1 to 8 h. RWC, relative water content; SMC, soil moisture content; FW, fresh weight; EL, electrolyte leakage.

To survey the variability in public drought data, we reanalyzed 109 water deficit RNAseq experiments in *Arabidopsis* obtained from the Sequence Read Archive (SRA). We manually curated metadata from 1,301 RNAseq samples across 109 BioProjects. These datasets include a comprehensive collection of genotypes and mutant backgrounds, developmental time points and tissues, and differences in stress severity and duration across a range of natural or controlled drought conditions (Fig. 1; Supplementary Table S1). Based on the available metadata, 81% of studies were conducted in growth chambers in standard potting media, 13% on agar plates, and 5% in greenhouses. Half of the studies (51%) applied an uncontrolled dry down by stopping irrigation, 27% had controlled drying to a set soil moisture content, 8% removed plants from media and let them air dry, and 14% used PEG to lower water potential and simulate water deficit responses. Surprisingly, 39% of *Arabidopsis* studies did not report paired physiological data such as photosynthetic metrics, leaf water potential, relative water content (RWC), osmotic adjustment, or indicators of membrane damage, all of which can provide critical information about drought severity and plant stress responses. Next, we processed the raw reads through a common pipeline to remove variation arising from the different algorithmic and statistical frameworks used in each individual study. Raw Illumina RNAseq reads were quality trimmed and aligned to the TAIR10 gene models, and raw or batch-corrected expression values in transcripts per million (TPM) were used as a basis for downstream analysis.

**Figure 2.**
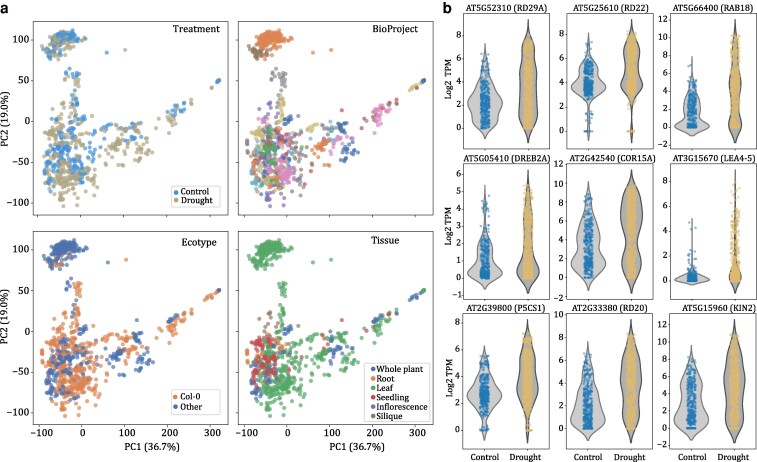
Dramatic variability of public drought gene expression datasets in *Arabidopsis*. (a) PCA of 1,301 drought-related RNAseq samples collected from the SRA. The first 2 principal components are plotted for all samples and each panel highlights a different experimental factor including a binary classification of drought and control (upper left), BioProject (upper right), genotype/accession of the sample (Col-0 or others; bottom left), and the tissue type (bottom right). Principal Components 3, 4, and 5 (not shown) explain 6.3%, 4.3%, and 3.5% of the variance, respectively. (b) Violin plot of log2-transformed TPM of RNAseq data for 9 drought marker genes in samples classified as control (left) and drought (right).

To identify any factors that clearly delineate samples within or across experiments, we used dimensionality reduction with an expectation that samples should cluster by water stress status. Principal component analysis (PCA) and t-Distributed Stochastic Neighbor Embedding (t-SNE) show no clear separation between drought-treated and control samples across *Arabidopsis* drought experiments (Fig. 2a; Supplementary Figs. S1 and S2). Within experiments, some BioProjects show clear separation of drought and control samples/replicates, but a surprising number have interspersed samples (Supplementary Fig. S3). Across experiments, samples were broadly separated by tissue type and BioProject. Root, seedlings, inflorescence, and siliques form groups in a similar dimensional space, whereas clusters of leaf and whole plant samples were more dispersed across PC1 and PC2 or Dimensions 1 and 2 for PCA and t-SNE, respectively (Fig. 2a; Supplementary Fig. S2). Samples within the same experiment or BioProject tended to cluster together, suggesting that the experimental effects are responsible for much of the separation we observed (Supplementary Fig. S3). Individual accessions of *Arabidopsis* show remarkable differences in expression dynamics under drought (Des Marais et al. 2012), and we tested if genotype differences may explain the lack of correlation between stressed samples across experiments. Similar to other experimental factors, there is no clear separation by ecotype (Fig. 2a). There is also no clear separation of samples by the reported duration, severity, or type of water deficit (eg, drying vs solute based) or technical variables such as sequencing read length, technology, read chemistry, or publication year.

To test if the observed separation by experiment (BioProject) rather than stress-related factors is caused by batch effects, we applied ComBat (Behdenna et al. 2023) to the expression matrix. This method leverages an empirical Bayes framework to estimate and subsequently adjust for batch effects. Post-ComBat adjustment, the first 2 principal components of the expression values accounted for only 19% of the total variance (Supplementary Fig. S4). However, even after controlling for BioProject, samples did not consistently separate by stress condition. This suggests that much of the variance removed by ComBat may include biologically meaningful differences confounded with the experimental batch, rather than purely technical artifacts. Together, this suggests drought experiments in *Arabidopsis* are highly variable and often difficult to compare across studies using traditional approaches.

We next sought to understand why the *Arabidopsis* drought RNAseq data appeared so variable across experiments. We first hypothesized that since dimensionality reduction provides a summary across all genes, individual factors such as drought stress may be confounded by other experimental factors. Therefore, to see if confounding factors are masking clustering of drought-stressed samples, we surveyed the expression pattern of 9 drought marker genes across all the samples. If confounding factors indeed masked the drought-response programs of relevant genes, we would expect the drought marker genes to be consistently induced in drought across all experiments. The 9 drought marker genes included RD29A (Nordin et al. 1991), RD22 (Yamaguchi-Shinozaki and Shinozaki 1993), RAB18, DREB2A (Liu et al. 1998), COR15A (Hincha et al. 2021), LEA4-5 (Bray 2004), P5CS1 (Yoshiba et al. 1995), RD20, and KIN2. Marker genes were generally expressed at higher levels in drought-stressed samples compared to well-watered, but this is highly variable across our datasets (Fig. 2b; Supplementary Fig. S5). For instance, the dehydrin RAB18 and ABA-induced transcription factor DREB2 were highly expressed under drought but generally not expressed under well-watered conditions. However, roughly a third of samples labeled as “drought” had no detectable expression of these 2 genes (Fig. 2b; Supplementary Fig. S5). This pattern is consistent across all 9 drought marker genes, suggesting the hypothesis that confounding factors are masking the drought response program is not supported. Instead, the data show that many drought-treated samples lack a molecular signature of water deficit and may not have experienced a physiologically relevant drought stress.

**Figure 3.**
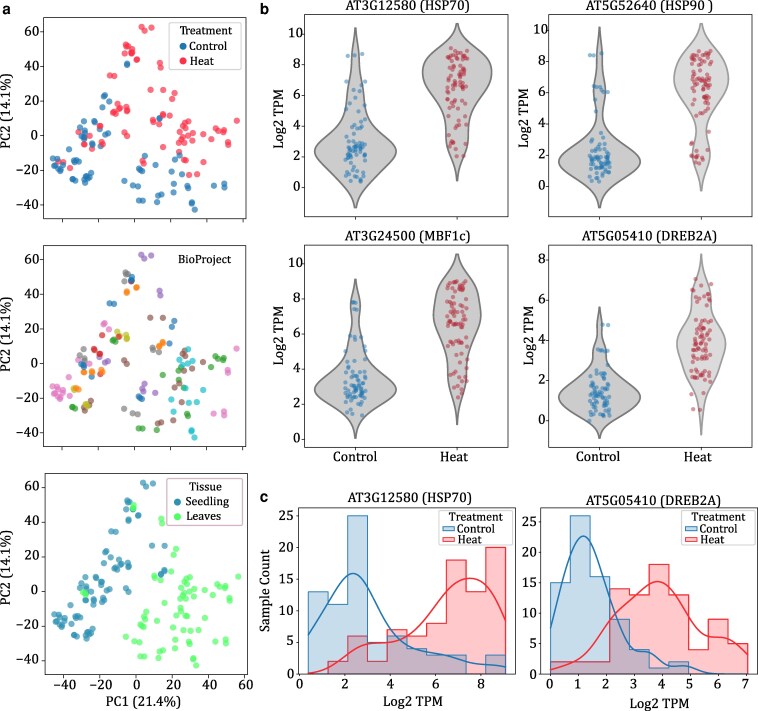
Comparison of public heat stress gene expression data in *Arabidopsis*. (a) PCA of 156 heat stress-related RNAseq samples collected from the SRA. The first 2 principal components are plotted for all samples and each panel highlights a different experimental factor including a binary classification of heat stressed and control (top), BioProject (middle), and the tissue type (bottom). (b) Violin plot of log2-transformed TPM of RNAseq data for 4 drought marker genes in samples classified as control (left) and heat stress (right). (c) Histogram of the same log2-transformed gene expression data as (b) for 2 heat stress marker genes of HSP70 (At3G12580; left) and DREB2A (AT5G05410; right).

We then wondered whether the observed inconsistencies in drought expression data might simply reflect a wide range of responses to various drought regimes applied, given the varying drought intensities and diverse growing conditions of different experiments. To understand if a similar variability exists in response to other stresses, we reanalyzed publicly available expression datasets related to heat stress in *Arabidopsis*. There are fewer published heat expression datasets, and we curated 21 experiments where plants were subjected to physiologically relevant heat stress in *Arabidopsis* between temperature ranges of 35 to 42 °C. Metadata indicated that growth conditions were similarly variable as drought studies, and the raw reads were processed as described above. Strikingly, when we performed dimensionality reduction on log-transformed expression data, the samples were distinctly categorized into either “heat” or “control” groups based on Principal Component 2, which explained 14% of the variation (Fig. 3a). Similar to the drought dataset, Principal Component 1, which explained 21% of the variation, differentiated the samples based on tissue type, grouping them as whole seedlings or leaves. Unlike in the drought dataset, there was no distinct separation by experiment (BioProject). We surveyed the expression patterns of 4 heat marker genes (HSP70, HSP90, MBF1c, and DREB2A) to see if individual genes have a similarly clear pattern. Heat marker genes have notably higher expression in all heat-stressed samples compared to control, and there is little overlap in the distribution of expression levels of these marker genes between the 2 conditions (Fig. 3b and 3c). Thus, public heat stress expression datasets demonstrate a clear molecular signature of heat stress. This signature persists through any experimental variation across studies, indicating that the inconsistencies noted in the drought stress data are not simply artifacts of work conducted in different laboratories.

### Developing a predictive model for classifying drought gene expression

Dimensionality reduction and clustering approaches were unable to delineate drought-stressed from control samples, and we hypothesize this was driven by quality issues of the underlying datasets. We previously developed a cross-species predictive model to accurately classify drought-stressed RNAseq data in maize and sorghum (Pardo et al. 2023) and sought to test if this approach could differentiate among drought and control samples in *Arabidopsis*. We developed random forest (RF)-based predictive models to classify the *Arabidopsis* samples as “drought” or “control” based on normalized gene expression values alone. We divided the RNAseq samples into a training set with 75% of the experiments (BioProjects) and a testing set with the remaining 25%. The overall accuracy of our predictive model was 66% (Supplementary Table S2), which is substantially lower than the model developed with high-quality maize and sorghum data. The precision and recall for “drought” samples were 0.79 and 0.51, respectively, and 0.61 and 0.84 for control. We tested 4 other classifiers including linear support vector classifier (SVC), simple neural network (MLP), histogram-based gradient boosting classifier (HGB), and K-nearest neighbor classifier (KNN) to see if this improved predictive accuracy. HGB had similar performance to RF (overall accuracy 65%) but SVC, KNN, and MLP performed worse with 52%, 52%, and 56% overall accuracy (Supplementary Table S2). The relatively low predictive accuracy in *Arabidopsis* was initially surprising, as our model was trained with significantly more data and tested within a single species with less genetic diversity than either maize or sorghum (Hufford et al. 2012, 2021; Boatwright et al. 2022; Igolkina et al. 2025). Further, *Arabidopsis* experiments are generally conducted within a narrower set of conditions compared to the growth chamber, greenhouse, and field environments for maize and sorghum. We suspect that the reduced predictive accuracy in *Arabidopsis* might be due to the inclusion of datasets with plants that are not physiologically stressed, thereby diminishing the effectiveness of our models.

**Figure 4.**
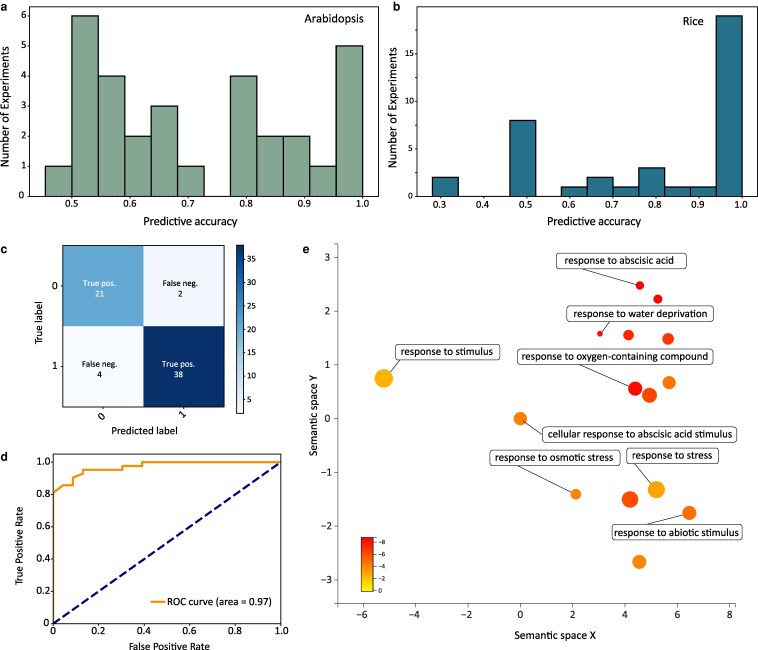
Predictive modeling of water stress status for drought expression data. Histogram of the predictive accuracy of the RF models for classifying drought using a leave-one-experiment-out approach for *Arabidopsis* (a) and rice (b). A predictive accuracy of 1 corresponds to perfect prediction, and 0.5 is more or less random. (c) Confusion matrix of the drought predictive model. (d) Receiver operating characteristic (ROC) curve showing the performance of the RF-based drought classification model across all classification thresholds. The dashed diagonal line indicates the performance of a random classifier. (e) Multidimensional scaling plot showing clusters of enriched GO terms for the top 100 most important features (genes) in the *Arabidopsis* RF machine learning models. The size of each circle is proportional to the number of genes annotated with each term, and the circles are shaded by the log10 of the adjusted *P*-value.

To assess the efficiency of the RF models in predicting drought and control samples for each *Arabidopsis* experiment, we employed the leave-one-group-out cross-validation method. This method involved iterative training of the model using all datasets except one and then gauging both its overall and individual sample performances. The aggregate performance was 0.71, with a precision of 0.77 and a recall of 0.75. Performance metrics for individual datasets varied widely, from a purely random prediction (approximately 0.5) to absolute accuracy (1.0) (Fig. 4a). RF had perfect or near-perfect prediction in 6 experiments, and 15 had 70% or higher predictive accuracy. We surveyed the expression patterns of drought marker genes for only these experiments and observed consistently higher expression in drought compared to control samples (Supplementary Fig. S6). This suggests that our predictive model is biologically relevant and that this subset of experiments were experiencing physiologically relevant drought stress.

Notably, 14 *Arabidopsis* experiments had performances nearing random results, with scores less than 0.65. Three of these experiments reported “mild” drought stress events where the authors collected limited physiology data to support the degree of plant stress (Clauw et al. 2015, 2016; Dubois et al. 2017). This includes 2 large-scale experiments of 6 and 98 *Arabidopsis* accessions subjected to “mild” drought stress in an automated plant phenotyping platform (Clauw et al. 2015, 2016). These experiments did not collect leaf water potential, making it difficult to assess the water status of the plants. To identify the most important underlying features in the model, we developed a RF classifier using training data from each *Arabidopsis* BioProject with a 70% or higher accuracy in the leave-one-group-out analysis. We reasoned that these datasets have the highest quality or most consistency and would be useful for developing an accurate predictive model. The overall accuracy improved to 91% with a 0.84 precision and 0.91 recall for control and 0.95 and 0.90 for drought, respectively (Fig. 4c and d). RF classifiers rank and quantify the importance of each feature in the underlying testing dataset, and we surveyed which features (genes) were most important for our drought predictive model. The top 100 genes with the most predictive power are enriched in Gene Ontology (GO) terms exclusively related to drought processes (Fig. 4e). This includes abscisic acid–activated signaling and responses to osmotic stress, water stress, oxygen-containing compounds, and ABA, among others. This is perhaps not surprising, but supports that our model is using genes with well-supported roles in drought responses to make its classification. Among the top predictors are regulators of the ABA signaling pathway (HAI1, HAI2, and ABI2), ABA-responsive proteins (RD29B, DIG2, and RAB1), LEA proteins (LEA4-5, ABR, and RAB18), and a lipid transfer protein involved in cuticle formation (LTP3) (Supplementary Table S3). The top predictors also include genes with unknown function (Supplementary Table S3), and predictive modeling may be used to identify new genes with uncharacterized roles in drought stress responses.

### Variability in drought expression profiles across crop and model plants

**Figure 5.**
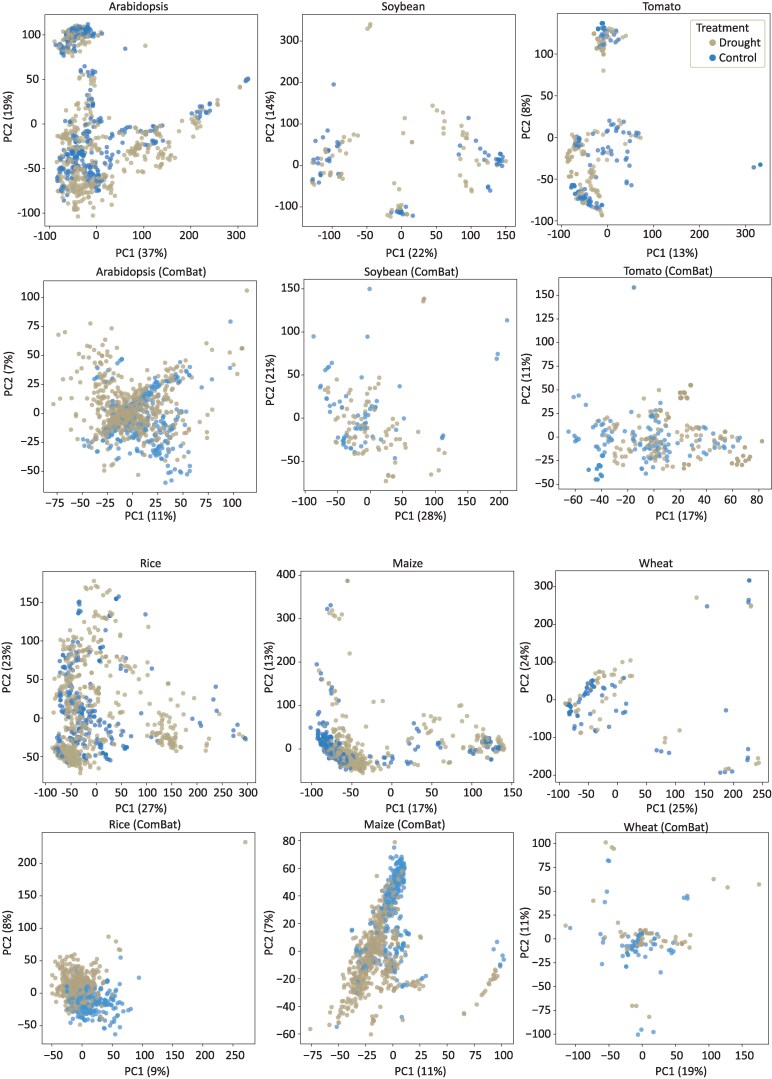
Variability of drought gene expression datasets across model and crop species. PCA plots are shown for drought gene expression datasets in the eudicots *Arabidopsis*, soybean, and tomato (top) and monocots rice, maize, and wheat (bottom). The PCA of *Arabidopsis* data from Fig. 2a is redrawn here to enable easier comparison. The first 2 principal components are plotted for all samples and separated into a binary classification of drought and control. Samples corresponding to recovery or unclear timepoints are not included. PCAs from log2 transformation of the raw TPMs are shown on the top panel for each species, and ComBat-corrected samples are shown below.

Our analyses suggested that drought RNAseq data in *Arabidopsis* is wildly variable, but is this a unique feature of *Arabidopsis* or a wider issue with drought studies in plants? To answer this question, we reanalyzed published RNAseq data for 5 additional plant species with between 12 and 57 individual drought experiments. This includes 179 soybean, 318 tomato, 137 wheat, 1,701 maize, and 981 rice RNAseq samples (Supplementary Table S1). Notably, these experiments exhibit greater variability compared to *Arabidopsis* in terms of genotypic diversity, tissue type, drought assay methods, environmental conditions (eg, greenhouse, growth chamber, or field settings), and stress severity. We processed this data using the same analytical pipeline that we applied to the *Arabidopsis* data, utilizing the most recent or highest-quality reference genome available for each species. Similar to the *Arabidopsis* results, PCA did not reveal clear distinctions between drought-exposed and control samples for any of the species (Fig. 5). This lack of separation could potentially be attributed to experimental artifacts, and to address this, we applied ComBat to mitigate batch effects across all of the BioProjects. The application of ComBat led to a reduction in variability among the studies for maize and rice, resulting in distinct clustering of drought and control groups in the adjusted expression data (Fig. 5). However, when applying ComBat to the wheat, soy, and tomato datasets, we did not observe a clear clustering pattern based on stress level, mirroring our observations in *Arabidopsis*. We tested leave-one-group-out cross-validation in rice to test if predictive models could more efficiently discern drought and control given the improved sample comparability compared to *Arabidopsis*. The aggregate performance across all models was 0.82, with a precision of 0.84 and a recall of 0.92. Performance metrics for individual datasets generally exceeded those observed in *Arabidopsis*, with 18 datasets achieving perfect predictive accuracy (Fig. 4b). Together, this suggests datasets outside of *Arabidopsis* profile drought more consistently and more comparable, but there is still variability.

## Discussion

Genome-scale datasets have increased exponentially, and millions of individual datasets from plants are publicly available. These datasets span the genomic, transcriptomic, epigenetic, and chromatin landscapes for thousands of diverse species aimed at numerous research questions related to plant form and function. Historically, most of these datasets were examined in isolation, often due to the specialized nature of each study and the lack of tools or interest in integrative analyses (Sielemann et al. 2020). However, recent computational and methodological advancements encourage data integration into more comprehensive comparative frameworks to identify conserved or emergent features of plant systems (Sreedasyam et al. 2023). A critical aspect of comparing multiple datasets is ensuring uniformity and consistency across experiments. Without this, the nuances of the data can become blurred, as sparsity and heterogeneity have the potential to overshadow crucial biological insights. Here, we reanalyzed the wealth of *Arabidopsis* drought gene expression data and found that inconsistencies across experiments, potential quality issues, and a lack of paired physiology data complicate integrative analyses. Below, we discuss the factors underlying these issues and propose guidelines that can enable enhanced comparability and reproducibility of future studies.

Applying consistent abiotic stress is relatively straightforward for heat, cold, salinity, UV, light, or nutrient deficiencies. Temperature can be raised or lowered for a set time, plants can be subjected to too much or too little light, and solutes or micronutrients can be maintained to predetermined molarities or concentrations to induce the desired deficiency. These discrete conditions enhance reproducibility across labs and enable cross-study comparisons. Our reanalysis of heat stress data in *Arabidopsis* supports this claim, as we identified a consistent expression profile under heat regardless of differences in growth conditions, developmental timing, tissue, or other variables across labs and separate studies. Drought stress, in contrast, is more difficult to apply, and the lack of standardization makes it difficult to compare experiments or even distinguish between control and stress samples in some cases. Drought stress itself is a complex and ill-defined stress with unclear delineations of mild and severe or tolerance and avoidance. More than other abiotic stresses, environmental contexts such as the volume and composition of soil or media, temperature, vapor pressure deficit, light intensity, and air flow affect the progression of drought stress. Plants growing in small pots, well-draining soil, higher temperatures, more light, or low humidity will dry faster. Days of drying are reported for most *Arabidopsis* studies, but this is an arbitrary metric that does not reflect discrete plant physiological states and is impossible to standardize across conditions without additional data to measure the water status of the plant and its soil. Even drought studies performed in the same environment can be challenging to standardize, as different genotypes or mutants can use water at different rates, causing individual plants to experience differing drought severities within the same timeframe and experiment (Ginzburg et al. 2022).

We observed that a surprising 39% of drought RNAseq experiments in *Arabidopsis* report no drought physiology data measuring plant water status, making it challenging to use these datasets in comparative studies or for meta-analyses. Many of these studies withheld watering for days to weeks and collected control and “drought”-stressed tissues for RNAseq and other downstream analyses without knowing the plant water status. Other studies measured only soil water content, which can indicate drying trends, but without paired physiological measurements from the plant at the time of sampling, it is difficult to assess whether the plants were actually experiencing drought stress. Consistent with this uncertainty, our reanalysis of large-scale studies that relied on soil moisture as the primary indicator of stress revealed no strong separation between drought and control treatments across genotypes. This suggests either subtle transcriptomic responses to the imposed drought or confounding genotype-by-environment interactions. These factors likely explain why we were unable to consistently separate stressed and control samples across studies, even after controlling for experimental artifacts and batch effects. In many cases, the gene expression profiles of “drought” samples closely resembled those of well-watered controls, suggesting that the plants may not have experienced, or perceived, a strong enough stress to elicit a robust transcriptional response. While gene expression alone does not define drought severity, in the absence of direct physiological measurements such as water potential, these transcriptomic similarities raise questions about the intensity or relevance of the applied stress. Conversely, it is also possible that some control samples were already experiencing mild or unintended stress, depending on the growth conditions used by different labs. For example, growth on agar plates and hydroponic media or in tightly packed pots can impose subtle water or nutrient stress even in well-watered plants, particularly under high light or low humidity conditions. Our machine learning models, which were trained primarily on datasets from moderate to severe stress in *Arabidopsis*, may have lacked sufficient training data from mild drought experiments and therefore struggled to classify samples from these studies accurately.

However, we emphasize that well-designed mild drought experiments with paired physiological data, particularly instantaneous measurements at the time of sampling, are critically needed to improve classification and better understand early or moderate drought responses. Removing datasets with poor performance significantly improved the accuracy of our predictive model to >90%, and we propose this model can be used as a quality control for future drought stress expression studies. Expanding the training dataset to include more experiments with gold-standard physiological measurements across a range of stress severities and environmental conditions will be essential for further improving model performance and for identifying robust gene expression signatures underlying drought stress responses.

Drought datasets outside of *Arabidopsis* are generally higher quality, with more consistent application of drought conditions and distinct signatures of water deficit responses. The reason for this is unclear. It is not difficult to stress *Arabidopsis*, and physiological signatures of water stress can be seen with a moderate decrease in water potential (−0.5 to 1.5 Mp). Compared to *Arabidopsis*, maize, rice, tomato, wheat, and soybean are generally grown at higher temperatures under higher light intensity, and they have much higher rates of evapotranspiration. When combined with greater plant biomass and the use of small pots, these species may experience drought stress after only a few days without watering in growth chambers and greenhouse settings. In contrast, small or uncrowded *Arabidopsis* plants may grow well for a week or more between waterings without experiencing water deficit (Ginzburg et al. 2022). Consequently, the typical protocol of withholding water for 7 to 10 d for *Arabidopsis* (see stress duration in Supplementary Table S1) may be insufficient for stressing plants, and this could explain the prevalence of lower quality datasets of *Arabidopsis* research.

Applying consistent drought is challenging, and efforts of standardizing stress severity have seen mixed success. Automated phenotyping systems can accurately manage soil water content, but they are expensive and have limited scale and flexibility, and plants are still subjected to other environmental fluctuations. Rainout shelters or controlled irrigation can help replicate natural drought conditions, but plants are often subjected to other environmental stresses throughout a growing season. Although there is no universally accepted method for conducting drought studies in plants, accurately quantifying water status and associated physiological responses can facilitate the comparison of experiments across different laboratories (Juenger and Verslues 2023). Developing a deeper understanding of drought responses requires integrating datasets that range from mild to sublethal and from a wide sampling of genotypes, tissues, and conditions. Rather than standardizing drought, we suggest that researchers should collect paired physiology, biochemistry, and morphological datasets at sufficient temporal and spatial resolution to quantify plant health. These traits can first verify that plants are experiencing the desired level of stress prior to expensive sequencing and serve as features or covariates for integration and reanalysis of multiple datasets.

## Materials and methods

### Assembling a representative catalog of gene expression RNAseq data

We assembled a database of drought RNAseq data in *Arabidopsis*, soybean, tomato, rice, maize, and rice from the NCBI SRA. Bulk data was retrieved using a series of drought- or heat stress-related keywords with the SRA Advanced Search Builder. The following metadata was collected for each experiment: tissue type(s), developmental stage, environment (eg, greenhouse, field, and growth chamber), media type, duration of stress, mechanism of drying, associated physiology datasets, genotype, number of timepoints, and number of replicates. Physiological metadata were classified as photosynthesis, gas exchange, stomatal conductance, chlorophyll fluorescence, and water status measurements (eg, RWC and water potential). One hundred twelve studies had a linked publication in the NCBI metadata, and 130 had no associated publication across all 6 species. Similar metadata was retrieved for individual SRA samples along with a binary classification of treatment (drought or control) where possible. Metadata was retrieved from the SRA and associated publications, but the lack of publications and ambiguity in some labels led to a high degree of missing or sparse metadata for many samples, and our manual annotations were conservative to reduce mislabeling samples for analysis and downstream predictive modeling. In this study, we define biological variability as the inherent differences in gene expression that arise from meaningful biological factors such as genotype, tissue type, developmental stage, and physiological response to drought. In contrast, technical variability refers to unintended differences introduced by experimental design or processing, including the method and timing of drought application, growth conditions (eg, pot size, soil type, and humidity), and molecular procedures such as RNA extraction and library preparation. These 2 forms of variability often co-occur and can be difficult to disentangle, particularly when physiological validation data are absent. Many of the studies we collected included knockout, overexpression, or gene-edited lines, and where appropriate, mutants were excluded from analysis and only wild-type control and drought samples were analyzed.

### RNAseq data processing

Raw RNAseq reads were downloaded from the NCBI SRA and quantified using a pipeline to trim, align, and quantify gene expression data (https://github.com/pardojer23/RNAseqV2). Briefly, sequence adapters were trimmed, and a quality check was performed on the raw FASTQ files using the fastp program (v0.23.2) (Chen et al. 2018). The cleaned sequencing reads were then pseudo-aligned to the *Arabidopsis* TAIR10 (Cheng et al. 2017), maize (*Zea mays* B73 V5) (Hufford et al. 2021), rice (*Oryza sativa Kitaake* v3.1) (Jain et al. 2019), tomato (*Solanum lycopersicum* ITAG4.0) (Hosmani et al. 2019), soybean (*Glycine max var. Williams* 82 V4) (Valliyodan et al. 2019), and wheat (*Triticum aestivum* cv. ‘Chinese Spring’ RefSeq v2.1) (Zhu et al. 2021) genomes using salmon (v1.6) (Patro et al. 2017). The transcript-level counts were converted to gene level using the R package TXimport (v 1.22.0) (Soneson et al. 2015). Raw TPMs or log2 + 1 transformed values were used for downstream analyses. The median alignment rate is 69.1% across all species and 70.8% in *Arabidopsis*, 79.5% in maize, 62.0% in rice, 65.1% in tomato, 64.7% in soybean, and 64.9% in wheat. These alignment rates are consistent with other meta-analyses of gene expression in *Arabidopsis* (Zhang et al. 2020). PCA was performed using built-in functions in scikit-learn (Pedregosa et al. 2011) on the log2-transformed gene expression data (TPMs) to reduce dimensionality and capture the main sources of variation within the datasets. The first 2 principal components were plotted for each species and labeled by various factors.

### Predictive modeling of drought stress responses

Our previous work on predictive modeling using drought gene expression in sorghum and maize found that the RF ensemble learning method performed best for classification (Pardo et al. 2023), so we first tested RF on our data. RF models were constructed using the RandomForestClassifier function from scikit-learn (v1.1.0) (Pedregosa et al. 2011). To select the hyperparameters, the RandomizedGridSearchCV function was utilized, with 100 iterations employing 3-fold cross-validation to traverse the parameter space. Samples were split into 75% training and 25% testing, and model performance was compared using the full unbalanced datasets for each species as well as balanced subsets. Analyses using balanced datasets in *Arabidopsis* had slightly lower but similar precision and recall, so the full set of samples were used. Feature importance was calculated using the mean decrease in impurity (Gini score) as implemented in scikit-learn (v1.1.0). All genes were subsequently ranked by their respective importance score. Enriched GO terms were calculated for the top predictive features in *Arabidopsis* using the Panther classification system (Mi et al. 2013).

We also tested predictive classification for 3 additional machine learning algorithms: linear SVC (LinearSVC), simple neural network (via the multilayer perceptron classifier, MLPClassifier), and HGB (HistGradientBoostingClassifier) implemented using scikit-learn (v1.1.0). The linear SVC was implemented using the LinearSVC class, and the fit method was used to train the model, and the predict method was applied to generate predictions. A simple neural network was developed using the MLPClassifier class. We initialized the MLP with 1 hidden layer of 100 neurons and trained the model using the fit method before making predictions with the predict method. The histogram-based gradient boosting classification tree, a variant of gradient boosting that is much faster than the traditional gradient boosting classifier, was implemented using the HistGradientBoostingClassifier class from the sklearn.ensemble module. This algorithm is capable of handling missing values, and it also applies the “early-stopping” method to avoid overfitting. For all the models, we evaluated their performance by calculating the accuracy score and generating a classification report, which included precision, recall, f1-score, and support for each class. RF outperformed other classifiers in all instances and was thus used for downstream analyses.

### Accession numbers

Sequence data from this article can be found in the BioProjects listed in Supplementary Table S1.

## Supplementary Material

kiaf653_Supplementary_Data

## Data Availability

The data analyzed in this meta-analysis are detailed in Supplementary Table S1, including SRA identifiers, PubMed IDs, and all other sample metadata. Raw expression values, expressed in TPM, are accessible on Dryad (https://doi.org/10.5061/dryad.7sqv9s50g). Jupyter notebooks containing all Python code used in this project, along with additional metadata, are available on GitHub: https://github.com/bobvanburen/Drought_meta_analysis_VanBuren_etal_2024.

## Supplementary material

The following materials are available in the online version of this article.

**Supplementary Figure S1.** Visualization of Arabidopsis drought gene expression data via t-SNE.

**Supplementary Figure S2.** Expanded PCA of Arabidopsis drought RNAseq data colored by BioProject.

**Supplemental Figure S3.** Principle component analysis of Arabidopsis drought data by individual experiment (BioProject).

**Supplemental Figure S4.** Principal component analysis of Combat adjusted Arabidopsis drought RNAseq data.

**Supplemental Figure S5.** Histogram of drought marker gene expression across Arabidopsis RNAseq data.

**Supplemental Figure S6.** Expression patterns of drought markers genes for only Arabidopsis experiments with high predictive accuracy.

**Supplementary Table S1.** Summary of the NCBI BioProjects analyzed in this project.

**Supplemental Table S2.** Precision and recall values for all classifiers tested using the Arabidopsis drought gene expression data.

**Supplemental Table S3.** Top features (genes) from the Random Forest based drought classifier model in Arabidopsis.

## Dive Curated Terms

The following phenotypic, genotypic, and functional terms are of significance to the work described in this paper:

KIN2 Gramene: AT5G15970
KIN2 Araport: AT5G15970

DREB2 Gramene: AT5G05410
DREB2 Araport: AT5G05410

RAB18 Gramene: AT5G66400
RAB18 Araport: AT5G66400

HSP70 Gramene: AT3G12580
HSP70 Araport: AT3G12580

ABI2 Gramene: AT5G57050
ABI2 Araport: AT5G57050

LTP3 Gramene: AT5G59320
LTP3 Araport: AT5G59320

RWC Gramene: Relative Water Content
RWC Araport: Relative Water Content

DIG2 Gramene: AT5G50360
DIG2 Araport: AT5G50360

root AmiGo: PO:0009005

SMC Gramene: Soil moisture content
SMC Araport: Soil moisture content
