# Supplementary material for: Variability in drought gene expression datasets highlights the need for paired physiology and community standardization
Source: Plant Physiol. 2025 Dec 16;200(1):kiaf653. doi: 10.1093/plphys/kiaf653 (PMC12854406; doi:10.1093/plphys/kiaf653)
Supplement: kiaf653_Supplementary_Data [file kiaf653_supplementary_data.zip › Supplemental_data_12-5-25.pdf]

Supplementary data VanBuren et al. 2025.

Supplemental Figures

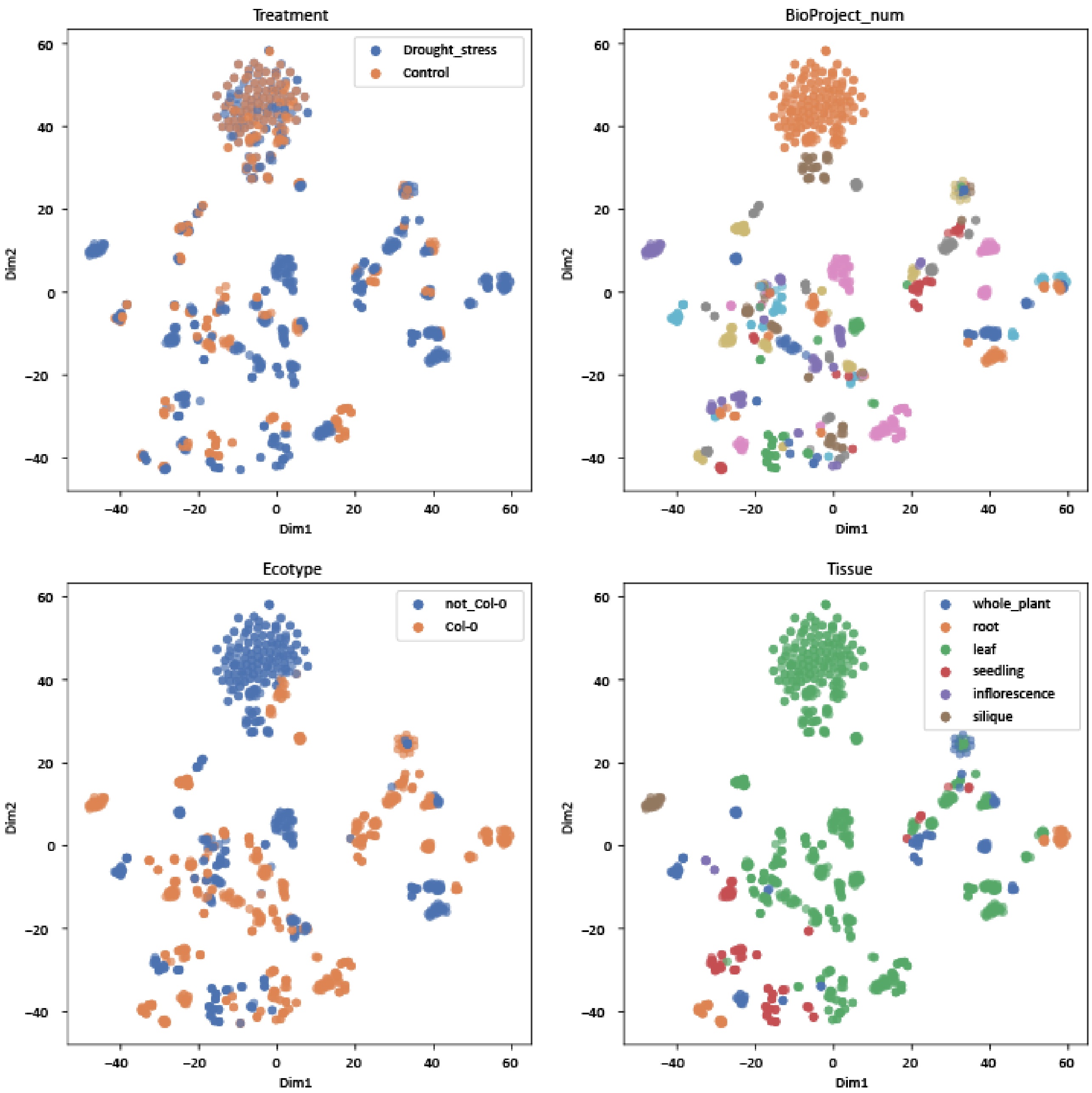

Supplemental Figure S1. Visualization of Arabidopsis drought gene expression data via t-SNE. This figure displays the first two dimensions of the t-SNE transformation applied to all samples and colored by different factors including a binary classification of drought and control (upper left), BioProject (upper right), genotype/accession of the sample (Col-0 or others; bottom left), and the tissue type (bottom right).

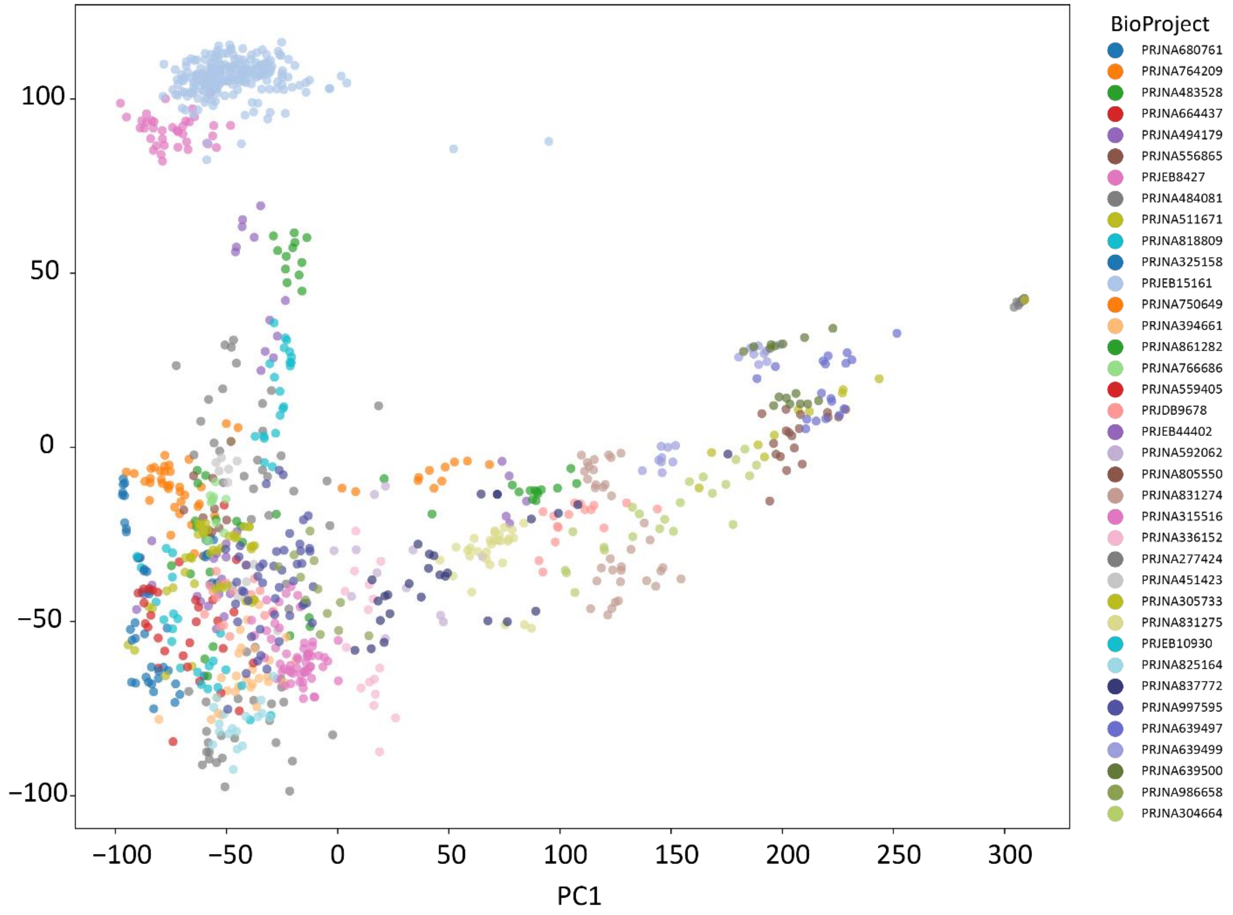

Supplemental Figure S2. Expanded PCA of Arabidopsis drought RNAseq data colored by BioProject. The data from Figure 2 is replotted and recolored, but only BioProjects with 12 or more samples are shown in this PCA for simplicity.

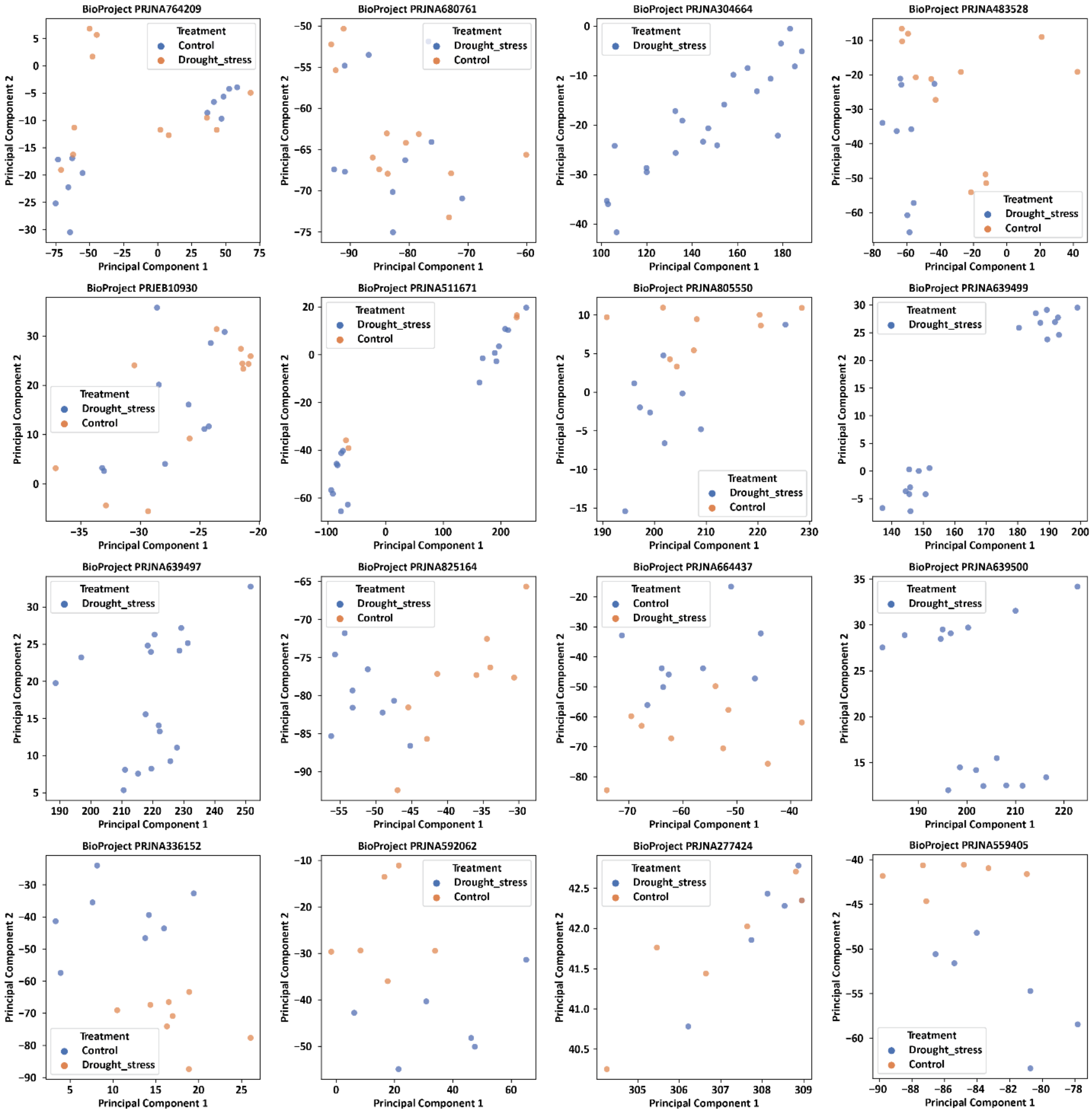
BioProject PRJNA764209
BioProject PRJNA680761
BioProject PRJNA304664
BioProject PRJNA483528
BioProject PRJEB10930
BioProject PRJNA511671
BioProject PRJNA805550
BioProject PRJNA639499
BioProject PRJNA639497
BioProject PRJNA825164
BioProject PRJNA664437
BioProject PRJNA639500
BioProject PRJNA336152
BioProject PRJNA592062
BioProject PRJNA277424
BioProject PRJNA559405
Principal Component 1
Principal Component 2
Treatment
Drought_stress
Control

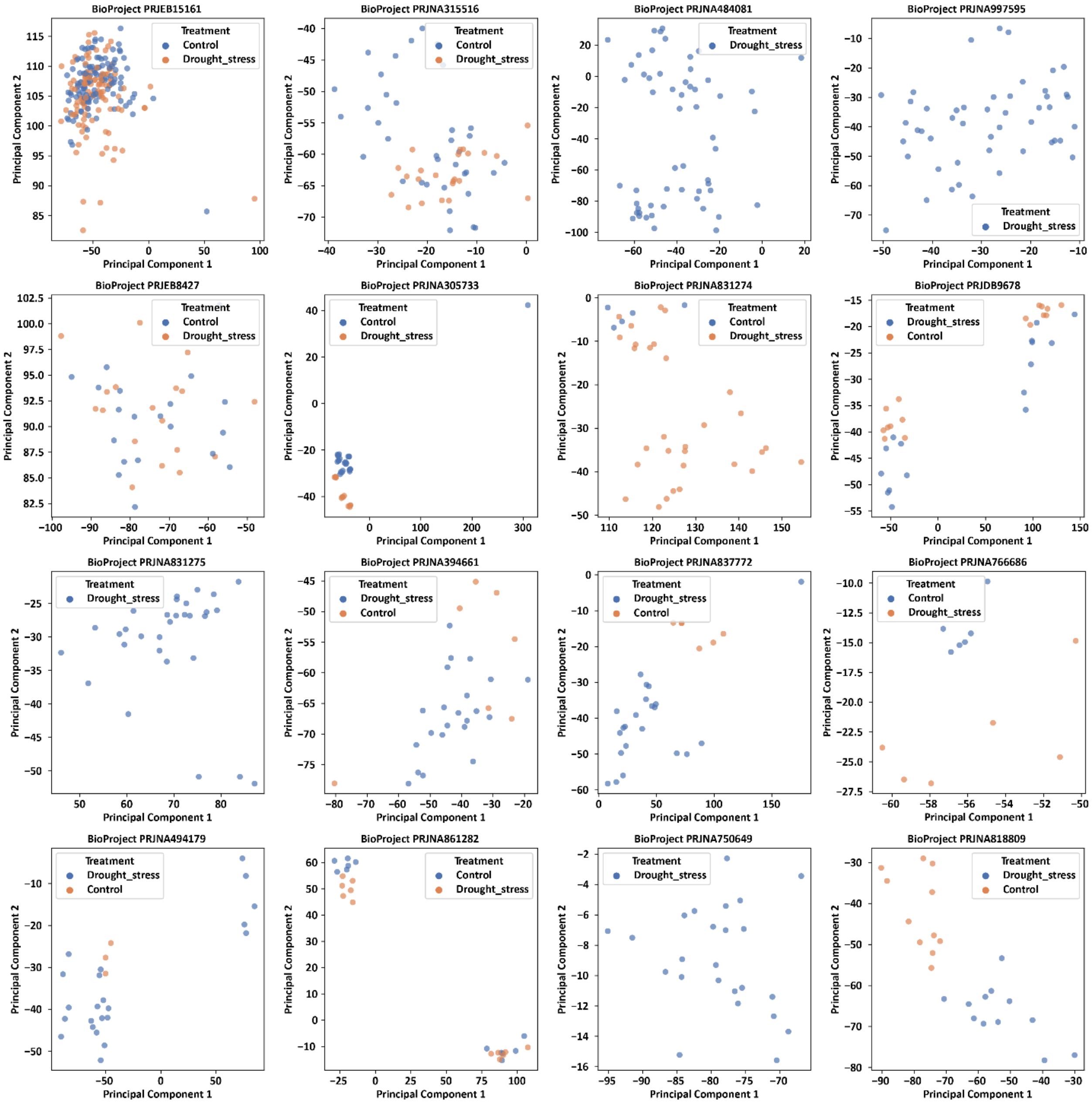

Supplemental Figure S3. Principle component analysis of Arabidopsis drought data by individual experiment (BioProject). Only experiments/BioProjects with 12 or more samples are shown.

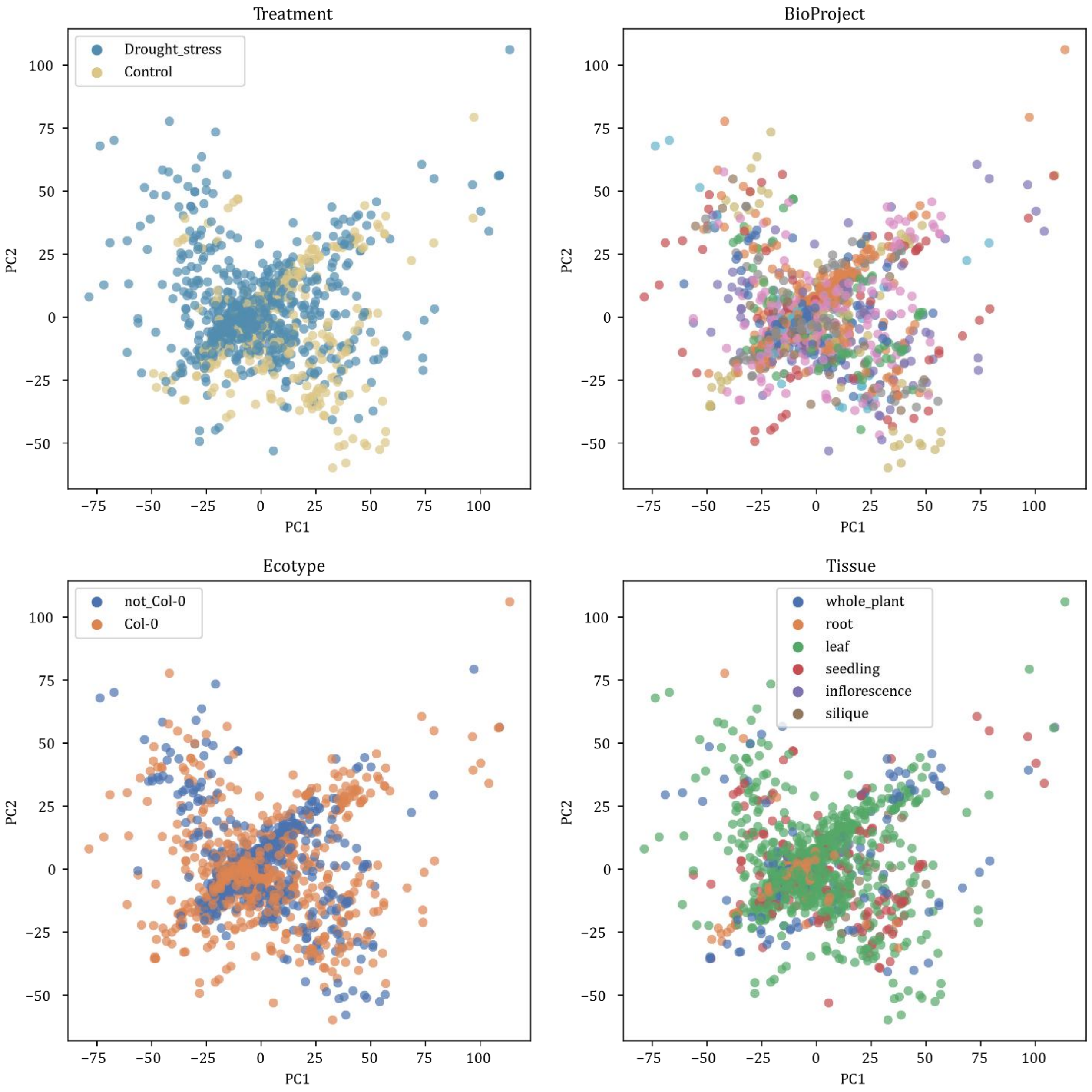

Supplemental Figure S4. Principal component analysis of Combat adjusted Arabidopsis drought RNAseq data. The first two principal components are plotted for adjusted expression values and colored by different factors including a binary classification of drought and control (upper left), BioProject (upper right), genotype/accession of the sample (Col-0 or others; bottom left), and the tissue type (bottom right).

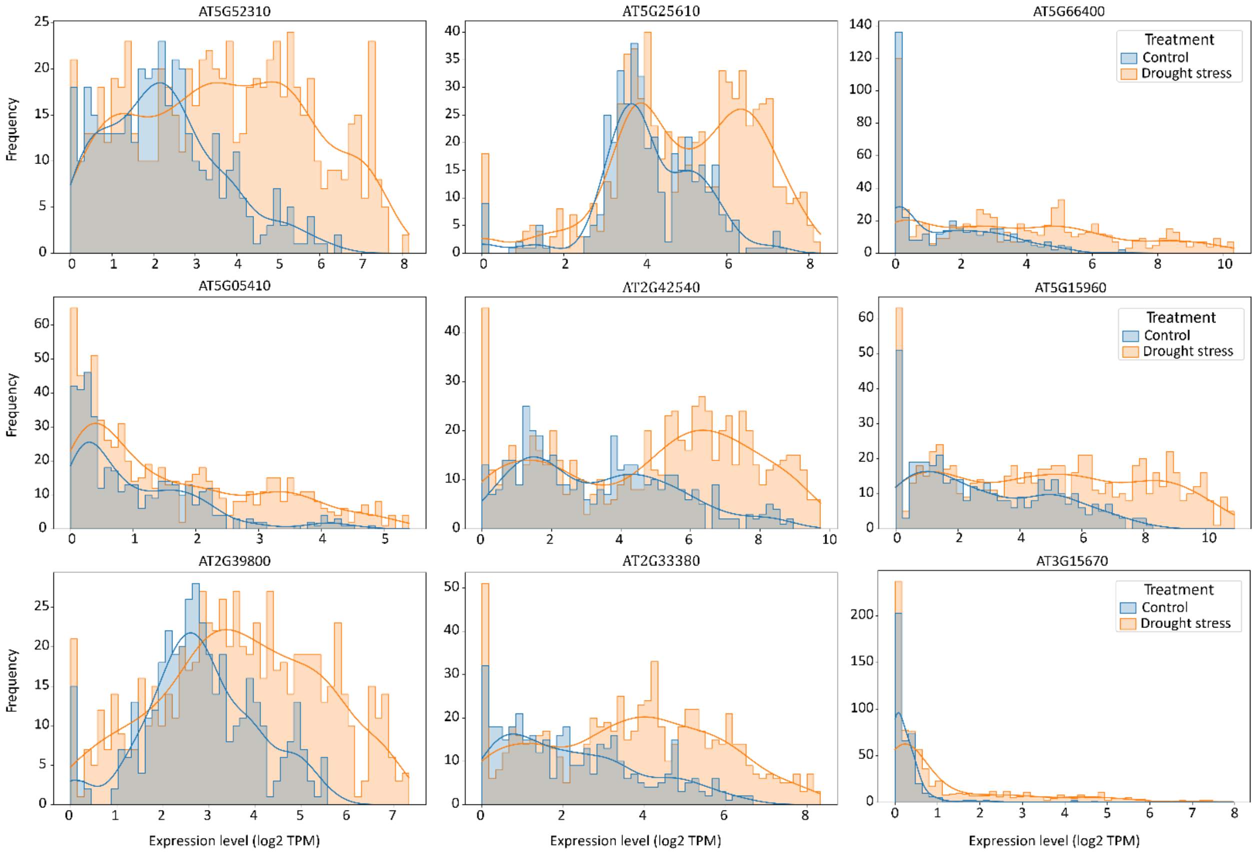

Supplemental Figure S5. Histogram of drought marker gene expression across Arabidopsis RNAseq data. Log2 transformed expression values are plotted for each drought (orange) and control (blue) sample for the classic drought marker genes shown in Figure 2.

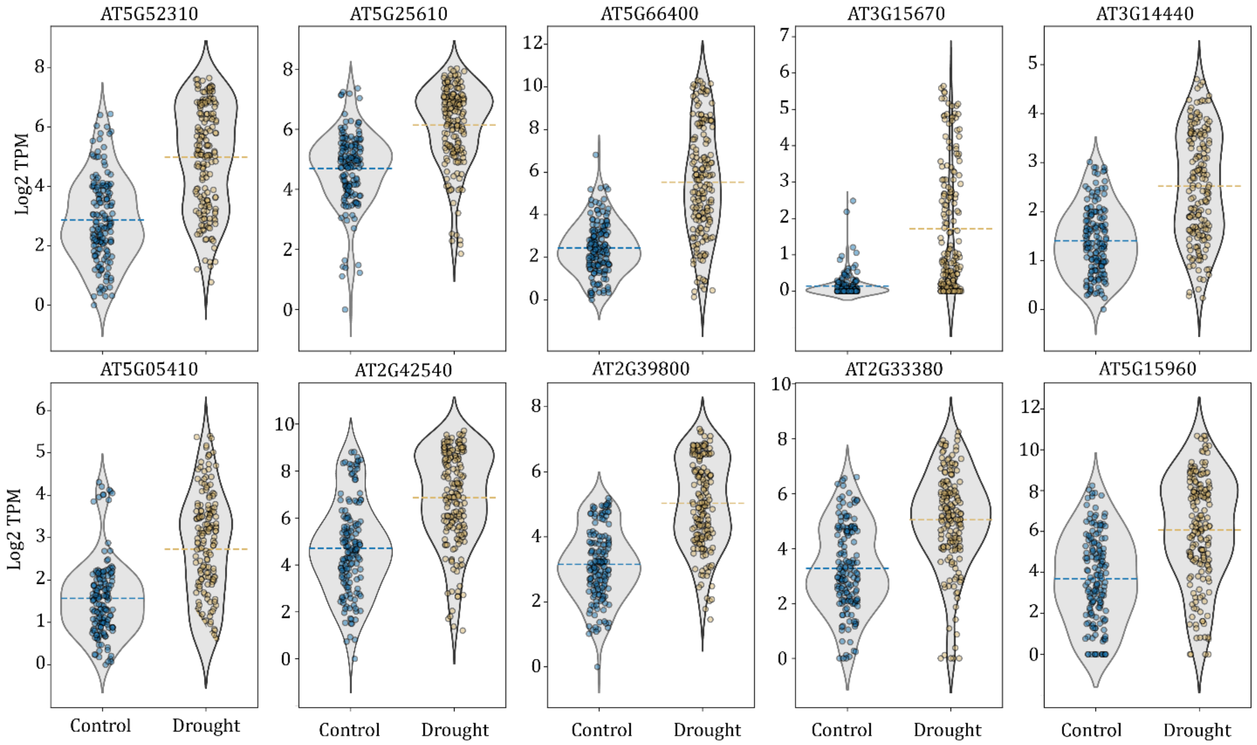

Supplemental Figure S6. Expression patterns of drought markers genes for only Arabidopsis experiments with high predictive accuracy.

Supplemental Tables

Supplemental Table S1. Summary of the NCBI BioProjects analyzed in this project. (see external excel file).

Supplemental Table S2. Precision and recall values for all classifiers tested using the Arabidopsis drought gene expression data.

Performance Metric	Random Forest	K-Nearest Neighbors	Linear Support Vector	Multi-Layer Perceptron	HistGradientBoosting
Precision (control)	0.6	0.49	0.49	0.52	0.59
Recall (control)	0.84	0.88	0.93	0.96	0.83
f1-score (control)	0.7	0.63	0.64	0.67	0.69
Precision (drought)	0.79	0.66	0.74	0.86	0.77
Recall (drought)	0.51	0.2	0.16	0.22	0.49
f1-score (drought)	0.62	0.3	0.26	0.36	0.6
**Overall accuracy**	**0.66**	**0.51**	**0.52**	**0.56**	**0.65**

Supplemental Table S3. Top features (genes) from the Random Forest based drought classifier model in Arabidopsis (see separate Excel file).
